# Untargeted flower volatilome profiling highlights differential pollinator attraction strategies in muscadine

**DOI:** 10.3389/fpls.2025.1548564

**Published:** 2025-02-28

**Authors:** Ahmed G. Darwish, Protiva R. Das, Eniola Olaoye, Pranavkumar Gajjar, Ahmed Ismail, Ahmed G. Mohamed, Violeta Tsolova, Nasser A. Hassan, Walid El Kayal, Kellie J. Walters, Islam El-Sharkawy

**Affiliations:** ^1^ Center for Viticulture and Small Fruit Research, College of Agriculture and Food Sciences, Florida A&M University, Tallahassee, FL, United States; ^2^ Department of Horticultural Sciences, Texas A&M University, College, Station, TX, United States; ^3^ Plant Sciences Department, University of Tennessee, Knoxville, TN, United States; ^4^ Department of Botany and Plant Sciences, University of California Riverside, Riverside, CA, United States; ^5^ Department of Horticulture, Faculty of Agriculture, Damanhour University, Damanhour, Egypt; ^6^ Synthetic Unit, Department of Photochemistry, Chemical Industries Research Institute, National Research Center, Cairo, Egypt; ^7^ Faculty of Agricultural and Food Sciences, American University of Beirut, Beirut, Lebanon

**Keywords:** floral aromas, muscadine flowers, marker volatiles, pollination attraction, volatilomics

## Abstract

Floral aromas are a mixture of volatile organic compounds, essential attributes associated with the attraction of different pollinators. This investigation is the first in-depth exploration of the volatile profiles of sixteen muscadine grape genotypes, producing female and perfect flowers using the headspace solid-phase microextraction (HS-SPME)-GC-MS-based untargeted volatilomics approach. A total of one hundred fifty volatile metabolites were identified in the muscadine flower genotypes, including the functional groups of hydrocarbons, esters, alcohols, ketones, aldehydes, miscellaneous, and acids. Multivariate statistical analysis for volatile terpenes revealed eleven bio-marker terpene volatiles that primarily distinguish between female and perfect flowers. The β-elemene, β-bisabolene, and α-muurolene were the marker volatiles characterizing perfect flowers; however, α-selinene, (*Z*,*E*)-α-farnesene, and (*E*,*E*)-geranyl linalool were the typical marker terpene in the female flowers. Perfect flowers exhibited better pollinator attraction capacity associated with a higher number of flowers per inflorescence, enhanced pollinator rewards, and higher numbers and quantities of terpene volatiles than female flowers, resulting in superior pollinator attraction capacity and fruit set efficiency. The pollinator attraction mechanism of female flowers exhibited several morphological and biochemical floral defects, causing random pollinator visits and low fruit set efficiency. The controlled pollination assay could express female flowers’ full fruit set capabilities by avoiding casual insect pollination. This comprehensive study suggests that these marker terpenes might contribute to pollinator attraction in muscadine flower genotypes and should be considered an excellent reference for agroecosystem ecologists and entomologists.

## Introduction

Over the past few decades, muscadine grapes have been getting increasing attention from consumers, growers, and breeders due to accumulating numerous human health functional metabolites, having distinct musky aromas, and producing unique flavors that are essential for processed beverage production and fresh market consumption (Darwish et al., 2021; Deng et al., 2022). *Muscadinia* is closely related to *Vitis* species that grow in the southeastern United States (Olien, 1990; Wen et al., 2018). Over 100 muscadine cultivars have been released in the southeastern region of the United States and extended to Chile and southern China (Wei et al., 2017; Hickey et al., 2019). Successful natural pollination is vital in flowering plants to achieve fruit production and seed development, thus maintaining ecological balance (Kearns and Inouye, 1997). The high efficiency and specificity of pollinator attraction are likely mediated through olfactory and/or visual cues, and there is increasing evidence that olfactory cues are paramount in attraction (Barragán-Fonseca et al., 2019). Muscadine grape flowers offer pollen and nectar as a reward to insect pollinators; however, pollen may be thrust upon them as calyptras eject (McGregor, 1976). The ability of insect pollinators to fertilize muscadine flowers has been associated with pollinator abundance and particular pollen-collection behavior. However, our knowledge about plant pollination on a biochemical level is still poorly understood (Mayer et al., 2011). Muscadine flower types include staminate (male), pistillate (female), or hermaphrodite (perfect). Pistillate vines produce large berries that are usually used for fresh consumption, in contrast, hermaphrodite vines produce smaller berries that are typically used for processed beverages (Campbell et al., 2021). Hermaphrodite flower muscadine genotypes are self-compatible and may be wind-pollinated. Conversely, wind plays a minor role in pistillate vines, where the majority of fruit set is attributed to insect pollination. Natural pollination in pistillate flower muscadines plays a crucial role in defining the yield; thus, lower pollination could result in less production due to a diminished fruit set (Sampson et al., 2001).

Floral volatile compounds greatly influence the attraction of pollinators and have been suggested as the critical mediator of plant-pollination networking (Dötterl and Vereecken, 2010; Dötterl and Gershenzon, 2023). These volatiles are highly variable among species regarding the disparities in their functional groups or due to differences in the absolute or relative amounts of compounds (Burkle and Runyon, 2019). Other biological studies have illustrated that floral volatiles serve additional functions, such as defending reproductive tissue against pathogens and attracting predators of plant pests (Boachon et al., 2019; Li et al., 2020). Floral volatiles are classified into a few primary functional groups according to their chemical structure and biosynthetic origin, such as fatty acid derivatives, terpenoids, phenylpropanoids, and benzenoids. Among all groups, floral terpenoids are the most dominant volatiles reported in horticultural crops and economically important plants (Dötterl and Gershenzon, 2023). Central floral volatile terpenes, including hemiterpenes (C5), monoterpenes (C10), sesquiterpenes (C15), and a few diterpenes (C20) are emitted into the air due to their high vapor pressures (Knudsen et al., 2006; Raguso, 2016; Pichersky and Raguso, 2018). Monoterpenes and sesquiterpenes are among the most abundant terpene components of floral scents and play crucial roles in plant development, chemical ecology, and pollinator attraction (Qiao et al., 2021; Dötterl and Gershenzon, 2023). For instance, sesquiterpenes, including (*E*)-β-caryophyllene, contribute to flower defense against bacterial pathogens (Huang et al., 2012). (*E*)-β-farnesene and (*E*)-α-bergamotene protect plants from microbial pathogens by recruiting their pollinators and pests predators. Similarly, the monoterpene linalool and its enantiomers attract bees and moths, enhance pollination in several flowering plants, and protect plants from microbial pathogens (Qiao et al., 2021). Flowers that produce the monoterpene (*E*)-β-ocimene are primarily attractive to honeybees and bumblebees (Crowell et al., 2002; Farré-Armengol et al., 2017). Recently, the identification of floral volatile profiles in flowering plants has increased rapidly due to advanced high-throughput analytical methods (Dötterl and Gershenzon, 2023). However, the volatile profiling of Vitaceae grape family flowers has not been substantially investigated. To our knowledge, only two reports are available on volatile analysis from grape (*Vitis vinifera*) flowers. Barbagallo et al. (2014) identified more than fifty volatiles from the flowers of several grape varieties while Gil et al. (2014) identified twelve volatiles that have a protective role against ultraviolet-B solar radiation and constitutive of the grape reproductive tissues. However, no reports are available on the volatile profiling of muscadine flowers.

Gas chromatography-mass spectrometry (GC-MS)-based untargeted volatilomics is currently the most utilized method to profile plant volatilome that allows a wide range of volatile compounds identification in different plant organs based on large reference libraries (e.g., NIST mass spectral library) and assessed for their discriminative impact (Pontes et al., 2009; Bryant and McClung, 2011; Bojko et al., 2014; Tieman et al., 2017). A study of GC-MS-based untargeted volatilomics analysis in strawberry flowers (*Fragaria × ananassa*) unveiled that the bee species *Bombus terrestris* or *Apis mellifera* showed strong responses to the floral compounds ethyl benzoate, (*Z*)-3-hexenyl propionate, (*Z*)-3-hexenyl acetate, benzeneacetaldehyde, and melonal (Liu et al., 2023). Another untargeted volatilomics study of twenty representative cucumber lines from various geographical locations revealed that 2-hexenal, 2,4-nonadienal, and 2,6-nonadienal are the key volatile metabolites that are relatively low in Korean cucumber lines, resulting in lower flavor intensity (Jo et al., 2022). Similarly, untargeted volatilomics analysis of forty-two citrus cultivars identified thymol derivatives, particularly *cis*-sabinene hydrate, sabinene, thymol, and thymol methyl ether, as the distinguishing marker volatile metabolites between citrus cultivars (Deng et al., 2022). Moreover, they showed that (*Z*)-3-hexen-1-ol contributes to the “green” aroma and also plays a role in distinguishing between orange and mandarin citrus groups.

In this study, untargeted volatile profiles of sixteen muscadine flower genotypes, including eight perfect and eight female flower vines, were characterized using HS-SPME-GC-MS-based analysis. Multivariate statistical analysis methods were utilized to determine the pivotal floral terpenoid volatiles, including principal component analysis (PCA), partial least-discriminate analysis (PLS-DA), and hierarchical heatmap analysis. Our results provide a foundation for further exploration of the functional characterization and evolution of marker volatile metabolites variation in muscadine flowers to understand their role in chemical ecology.

## Materials and methods

### Plant materials and experimental conditions

**Figure 1:**
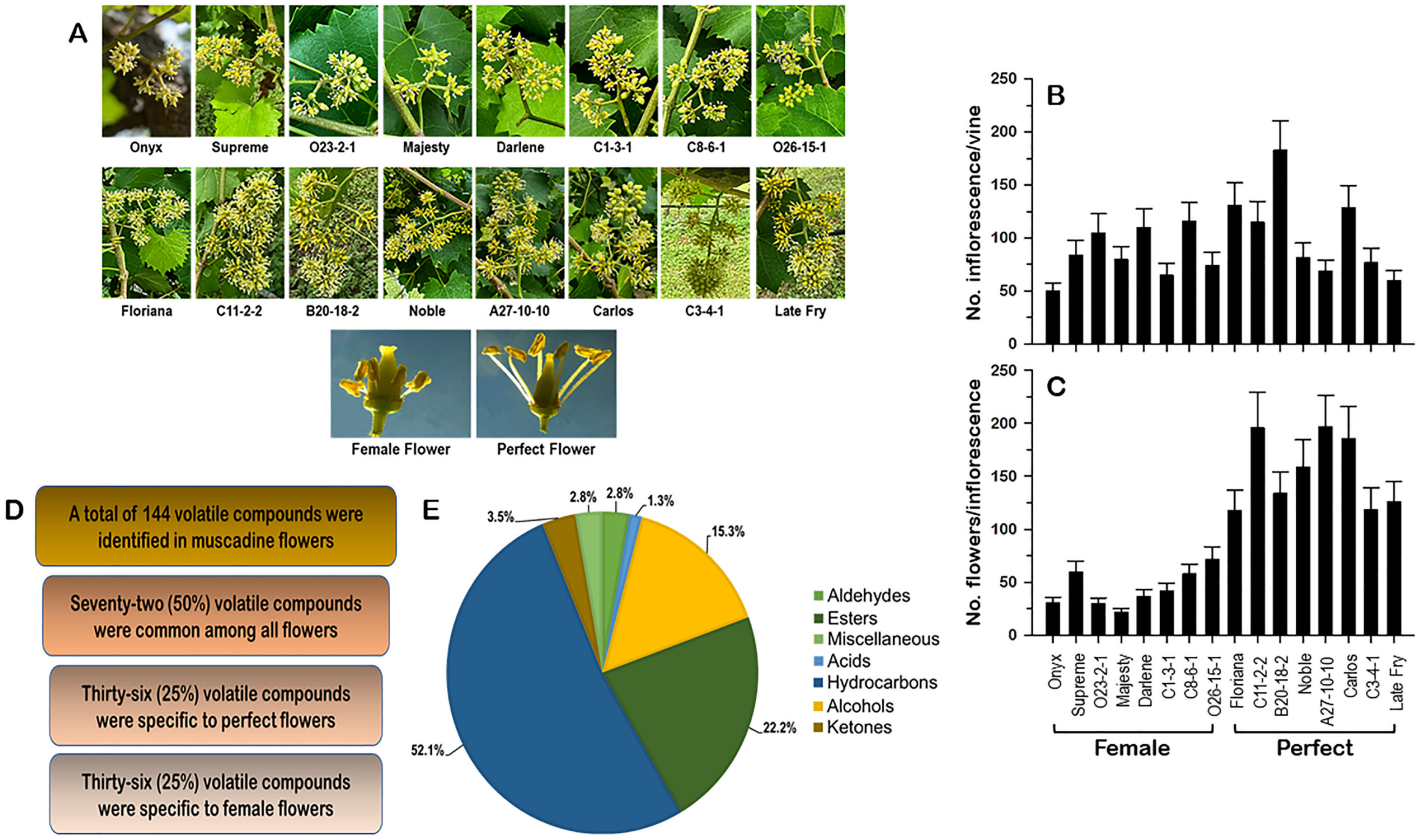
**(A)** Close-up views of the female and perfect muscadine flower genotypes used in this study, defining the differences in the inflorescence and flower structures. The characterization of the number of inflorescences/vine **(B)** and the number of flowers/inflorescence **(C)** of muscadine genotypes used in this study. **(D)** Number of identified volatile compounds in female and perfect muscadine flowers. **(E)** Pie chart of volatile functional groups identified in muscadine flowers.

Sixteen muscadine grape genotypes (*Muscadinia rotundifolia* Michx.), including eight standard cultivars and eight breeding lines, were used in this study (**Supplementary Table S1**). All muscadine vines were grown at the Center for Viticulture, Tallahassee, Florida (30°28′45.63′′ N, 84°10′16.43′′ W). Vineyard management and practices followed the guidelines outlined in the Muscadine Production Guide for Florida written by the Center for Viticulture and Small Fruit Research (CVSFR), Florida Agricultural & Mechanical University (FAMU) (https://famu.edu/viticulture). The breeding lines were developed under the grape-breeding program of the CVSFR at FAMU (Tallahassee, FL, USA). The muscadine genotypes were selected according to the diversity in their flower type, perfect (hermaphrodite) and female (pistillate) that produce bronze and red berries (**Figure 1A**). Samples were collected from 15-year-old grapevines at the open flower stage. The flower buds were carefully separated from the flower cluster, and samples were randomly assembled in three biological replicates. All samples were immediately frozen in liquid nitrogen and stored at −80°C for further analysis.

### Muscadine sample preparation for HS-SPME

The floral volatile compounds (VOCs) from muscadine flowers were assessed using HS-SPME. Frozen samples were lyophilized and ground into a fine powder using a Geno/Grinder 2010 (Metuchen, NJ, USA). A 200 mg powder was mixed with 20 μl of 2-octanol and transferred to a 25 ml glass vial (Thermo Scientific, Bellefonte, PA, USA). A 5 ml of saturated sodium chloride (NaCl) solution was added to inhibit enzyme degradation during extraction. All samples were incubated with a magnetic stirrer to facilitate VOC release before the glass vial was capped. The homogenized samples were incubated for 30 min in a 60°C water bath with continuous agitation (vortex every 5 min). Afterward, the VOCs were collected using a 2 cm DVB/CAR/PMDS SPME fiber (50/30μm, Supelco Inc., Bellefonte, PA, USA) by exposing the fiber to the headspace for another 30 min under the same conditions. The fibers were activated before sampling according to the manufacturer’s instructions. After the incubation step, the SPME fiber was inserted directly into the injection port of the GC system for thermal desorption (4 min at 250°C) in a splitless mode.

### Gas chromatography-mass spectrometer analysis

GC-MS analysis was performed using a Shimadzu-QP2010SE (Shimadzu Scientific Instruments, INC, Columbia, MD 21046, USA) coupled with an SH-Rxi-5Sil MS column (30 m x 0.25 mm i.d., film thickness 0.25 μm). The column oven temperature program was initiated at 40°C for 4 min, then ramped up to 245°C at a rate of 5°C min^−1^ and held for 5 min. The SPME fiber was inserted manually into the GC-MS apparatus operating in EI mode at 70 eV. The transfer line, ion source, and quadrupole mass detector temperature values were set to 250°C, 230°C, and 150°C, respectively. The carrier gas (Helium) flow rate was 0.8 ml/min using splitless mode. The relative percentage quantity of each component was calculated by comparing its average peak area to the total area. MS solution software provided by the supplier was used to control the system and to acquire the data.

### Identification and quantification of floral volatiles

The peaks obtained from GC-MS (**Supplementary Figure S1**) were first processed using Shimadzu software (Shimadzu Scientific Instruments, INC, Columbia, MD 21046, USA). The parameters used for raw peak extraction, data baseline filtering and calibration of the baseline, peak alignment, deconvolution analysis, peak identification, integration, and spectrum match of the peak area were the same for all samples. VOCs were identified by matching their retention indices (RI) and mass spectra with those available from the National Institute Standard and Technology (NIST) database (Gaithersburg, MA, USA) and Wiley libraries. Relative amounts of the identified VOCs were calculated from the total ion chromatogram (TIC). Correspondingly, the peak area of each VOC was converted into a relative concentration value.

### Pollination assay

Five female and perfect vines of muscadine cultivars ‘Darlene’ and ‘Floriana’, respectively, were selected for pollination assay. All vines were 15 years of age and planted in a 53-muscadine cultivar trial block. For each vine, 20 inflorescences at a similar developmental stage and size were randomly selected and separated into two groups of 10 inflorescences used for controlled and open pollination. The flowers designated for controlled pollination of the perfect genotype ‘Floriana’ were subjected to an emasculation procedure to prevent potential self-pollination. All controlled pollination inflorescences were bagged and checked daily for readiness for pollination. Pollination began when about 20% of the flowers opened and continued every morning until stigmas showed dryness typical of post-pollination, which usually lasted a week. Fresh flowers from ´Granny Val´ were directly applied to the female ‘Darlene’ and emasculated ‘Floriana’ flowers. Pollinated inflorescences remained in bags until the fruit set was completed. After harvest, all clusters were collected and separated based on vine, cultivar, and type of pollination assay (open or controlled). All clusters were evaluated for the traits of cluster weight, weight of berries per cluster without the rachis, number of berries per cluster, and individual berry weight. All records were the average of five values (± standard deviation).

### Multivariate statistical analysis

All multivariate statistical analyses, including principal components analysis (PCA), partial least-squares–discriminant analysis (PLS-DA), and heatmap of Pearson correlation analysis, were performed using MetaboAnalyst 6.0 online software. The data were normalized into a logarithmic base for statistical analysis, and Autoscaling was performed. PCA was first used as an unsupervised method to determine whether there were fundamental differences among muscadine flower genotypes. Furthermore, supervised regression modeling was performed on the data set using PLS-DA to obtain the variable importance in the projection (VIP). The marker volatile metabolites were filtered and confirmed by combining the results of the VIP, |p|, and |p(corr)|, and the screened volatile compounds were analyzed by the heatmap of Pearson correlation with flower genotypes to characterize key volatile metabolites variation among genotypes. Figures were generated using Prism (GraphPad Prism 5.01Inc. La Jolla, CA, USA) statistical software.

## Results and discussion

### Muscadine flower characteristics

Grapevine flower traits, such as the number of inflorescences per vine and the number of flowers per inflorescence, provide crucial insights for assessing yield potential. From a quantitative standpoint, a higher number of inflorescences and flowers per vine enhances the floral signal display, increases the quantity of volatile organic compounds (VOCs), and improves floral rewards, which collectively optimize pollinator attraction and fruit set efficiency (de Ibarra et al., 2015). In this study, sixteen muscadine grape genotypes were analyzed, with eight producing female (pistillate) flowers and eight producing perfect (hermaphrodite) flowers (**Figure 1A**; **Supplementary Table S1**). Muscadine inflorescences are typically small and consist of tiny green flowers. To assess each vine genotype’s ability to attract pollinators, the number of inflorescences per vine was counted (**Figure 1B**). The number of inflorescences varied widely depending on the genotype, independent of flower type (female or perfect). The average number of inflorescences across all genotypes was 95.6 ± 8.5. The female cultivar ‘Onyx’ had the lowest inflorescences number (50 ± 7.5), while the perfect-flowered genotype ‘B20-18-2’ exhibited the highest number (183 ± 27.5). Similarly, the number of flowers per inflorescence showed significant variation among genotypes, with a clear distinction based on flower type (**Figure 1C**). Female flower genotypes had fewer flowers per inflorescence (44 ± 6.2), with ‘Majesty’ having the lowest count (22 ± 3.3) and ‘O26-15-1’ the highest (72 ± 9.6). In contrast, perfect-flower genotypes exhibited a higher average flower count per inflorescence (168.6 ± 10.5). Among these genotypes, ‘Floriana’ had the lowest average, with 118 ± 18.9 flowers per inflorescence, while ‘A27-10-10’ boasted the highest average, reaching 197 ± 29.6 flowers per inflorescence.

The number of inflorescences per vine and the number of flowers per inflorescence are valuable parameters for pre-evaluating the yield. The inflorescences of female muscadine vines displayed a weaker morphological phenotype. The floral signal is smaller than in perfect flower and the number of flowers in the inflorescence is lower, negatively affecting the efficiency of their interaction with pollinators. The attractiveness of muscadine flowers for pollinators is largely determined by VOCs and the quality of floral rewards in the form of nectar and pollen (Knauer and Schiestl, 2015; Cane, 2016; Delle-Vedove et al., 2017). This preliminary assessment suggested that perfect flowers are endowed with an absolute advantage in terms of all floral traits, affecting pollinators’ attraction and the ability to set fruit.

### Volatile profiles of muscadine flowers

Volatile organic compounds (VOCs) are essential in attracting pollinators, boosting yield and quality, and protecting flowers from pathogens (Bouwmeester et al., 2019). In this study, untargeted volatile profiling using HS-SPME-GC-MS, coupled with NIST mass spectral library identification, highlighted significant differences between female and perfect muscadine flowers. A total of 144 volatile metabolites were identified across muscadine flower genotypes (**Supplementary Table S2**). Among them, 25% were specific to female flowers, while another 25% were exclusive to perfect flowers. The remaining 50% were shared between both flower types (**Figure 1D**). The identified VOCs predominantly consisted of hydrocarbons (52.1%), followed by esters (22.2%) and alcohols (15.3%). Smaller proportions were contributed by ketones (3.5%), aldehydes (2.8%), miscellaneous compounds (2.8%), and acids (1.3%) (**Figure 1E**, **Supplementary Table S2**). These results are consistent with the known VOC profiles of other floral species (Dötterl and Gershenzon, 2023).

The total VOC emissions varied widely among muscadine genotypes, with perfect flowers producing nearly double the amount of VOCs compared to female flowers. Total VOC levels ranged from 417.4 ± 66.3 µg/100g in the female cultivar ‘Darlene’ to 8632.7 ± 1294.9 µg/100g in the perfect-flowered ‘Late Fry’ (**Supplementary Table S2**). Overall, female flowers emitted a significantly lower number of volatile compounds (35.3 ± 3.3) compared to perfect flowers (45.9 ± 3.1). Among examined muscadine genotypes, ‘Darlene’ produced the fewest volatile compounds, with only 21 identified, while ‘Late Fry’ yielded the highest number, with a total of 65 volatiles. The diversity, quantity, and functionality of these VOCs are critical factors in attracting pollinators (Burkle and Runyon, 2016). These findings suggest that perfect muscadine flowers, which emit higher levels and numbers of VOCs, may have an ecological advantage in reproductive success due to enhanced pollinator attraction.

### Muscadines’ floral alcohol volatiles

The diversity and level of alcohols in floral scents vary across species and even between genotypes within the same species, influencing pollinator preferences and specialization (Pichersky and Gershenzon, 2002). In the muscadine flowers, 22 alcohols were identified, with distinct differences in their distribution between female and perfect flowers. Of these alcohols, seven (31.8%) were specifically emitted by female flowers, and three (13.6%) were exclusively detected in perfect flowers, suggesting that these alcohol volatiles may serve specific roles depending on the flower type (**Supplementary Table S2**). However, twelve alcohols (54.6%) were present in both flower types, indicating commonalities in their function across female and perfect flowers, likely contributing to shared characteristics of muscadine flower fragrance. The average number of alcohols produced per genotype was 6.1 ± 0.5. The female ‘O23-2-1’ flowers had the highest number of alcohols (11), while the perfect ‘Noble’ flowers produced the lowest (3), illustrating significant variation in alcohol production across different flower types and genotypes.

The total alcohol production in muscadine flowers also varied widely across genotypes. The lowest alcohol content was observed in the female ‘Darlene’ flowers (40.7 µg/100g), while the highest was found in the perfect ‘Late Fry’ flowers (2115.6 µg/100g). Interestingly, these two genotypes also represented the extremes in total VOC content, underscoring the considerable contribution of alcohols to the overall volatile profile. The predominant alcohol detected in female flowers was (6*E*)3,7,11-trimethyl-1,6,10-dodecatrien-3-ol. In contrast, no specific alcohols were identified in perfect flowers. However, several alcohols, including 1-hexanol, 1-octanol, 1-decanol, (*E*)-2-octen-1-ol, and 2,3,6-trimethyl-7-octen-3-ol, were common to both flower types, highlighting some overlap in their volatile profiles (**Supplementary Table S2**).

Alcohols are vital components of floral VOCs, playing key roles in pollinator attraction, plant defense, and ecological communication. They contribute uniquely to the floral scent bouquet, influencing a plant’s reproductive success by attracting specific pollinators. For instance, (6*E*)3,7,11-trimethyl-1,6,10-dodecatrien-3-ol, with its sweet, floral aroma, attracts moths and bees (Raguso and Pichersky, 1999). Other alcohols, like 2,3,6-trimethyl-7-octen-3-ol, add fruity notes, while 1-hexanol provides a fresh, green scent (Pichersky and Gershenzon, 2002; Dudareva et al., 2006; Knudsen et al., 2006). Complex floral fragrances result from synergistic interactions between alcohols and other VOCs, enhancing pollinator attraction through nuanced scent profiles (Dudareva et al., 2006; Knudsen et al., 2006). Beyond attracting pollinators, alcohols like 1-decanol and (*E*)-2-octen-1-ol play defensive roles, signaling beneficial insects and protecting plants from herbivores and pathogens. For example, 1-decanol has antimicrobial properties that support plant defense, while 1-octanol and (*E*)-2-octen-1-ol attract natural enemies of herbivores, contributing to plant fitness and survival (Kessler and Baldwin, 2001; Pichersky and Dudareva, 2007; Park et al., 2008; Schiestl, 2010). In summary, alcohols are crucial in shaping floral scents, attracting pollinators, defending plants, and facilitating ecological interactions, highlighting their significance in plant ecology and evolution.

### Muscadines’ floral ester volatiles

In muscadine flowers, 32 volatile esters were identified, with five (15.6%) found only in female flowers and eight (25%) exclusively in perfect flowers. The remaining 19 esters (59.4%) were common to both flower types. In general, perfect flowers released a considerably higher number of volatile esters (11 ± 0.7) when compared to female flowers (8.4 ± 0.9). The female ‘C8-6-1’ genotype exhibited the lowest number of esters, producing only five, while the perfect ‘Late Fry’ flowers generated the most, with 15 esters. The total quantity of esters emitted by muscadine flowers varied significantly, ranging from 35.5 ± 5.3 µg/100g in the female genotype ‘C8-6-1’ to 2961.8 ± 473.9 µg/100g in the perfect cultivar ‘Noble’ (**Supplementary Table S2**). On average, perfect flowers emitted ~2.9 times more ester volatiles than female flowers. The dominant esters in perfect flowers included methyl stearidonate, *cis*-9-Tetradecenoic acid, propyl ester, and butyl myristate, while no unique esters were identified for female flowers. However, esters such as octyl formate, butyl dodecanoate, *Z*-5,17-octadecadien-1-ol acetate, methyl tetradecanoate, octyl octanoate, 2-*O*-(2-ethylhexyl) 1-*O*-tridecyl oxalate, and 2-ethylhexyl pentyl sulfite were detected in both flower types (**Supplementary Table S2**).

Volatile esters play essential roles in flower odors by contributing to pollinator attraction and enhancing scent complexity. These compounds are crucial in determining the floral fragrance profile via shaping specific scent signals. For example, esters like *Z*-5,17-octadecadien-1-ol acetate and 2-*O*-(2-ethylhexyl) 1-*O*-tridecyl oxalate may contribute to species-specific scent signatures. They are often detected in particular plant species and serve as critical components in distinguishing the scent profiles of those species, ensuring that pollinators can easily locate their preferred flowers (Verdonk et al., 2003). Other esters like octyl formate and methyl tetradecanoate enhance the complexity of floral scents and play a role in plant ecological communication (Pichersky and Gershenzon, 2002; Pichersky and Dudareva, 2007). They add fruity and floral notes to flowers, increasing their appeal to diverse pollinators (Knudsen et al., 2006; Dudareva et al., 2013). Finally, the unique and synergistic interaction of esters with other VOCs makes them essential in ensuring effective plant-pollinator interactions. Esters such as octyl octanoate and butyl dodecanoate interact synergistically with alcohols, terpenes, and aldehydes to refine and enhance floral odors. These interactions are crucial for developing complex floral scent profiles that can appeal to different types of pollinators (Dudareva et al., 2004, 2013).

### Other minor volatile compounds

The volatilome profiling of muscadine flowers revealed the presence of several minor volatile compounds, including aldehydes, ketones, miscellaneous compounds, and acids.

### Aldehydes

Four aldehyde volatiles were identified in muscadine flowers (**Supplementary Table S2)**. The total aldehyde levels produced by muscadine flowers varied significantly among genotypes, with a range of 210.2 µg/100g, from a minimum of 4.8 ± 0.8 µg/100g in female flowers of the ‘Supreme’ cultivar to a maximum of 215 ± 32.3 µg/100g in female flowers of ‘Majesty’. On average, perfect flowers emitted ~2.5 times more aldehydes than female flowers. Aldehydes are essential contributors to floral scents, playing key roles in pollinator attraction and plant defense (Surburg and Panten, 2005). They are also involved in the biosynthesis of aroma-volatile esters, interconverting them into alcohols and serving as substrates for ester formation (El-Sharkawy et al., 2005; Manríquez et al., 2006). In muscadine flowers, aldehydes contribute to unique odor profiles and ecological functions. For example, (*E*)-2-hexenal provides a sharp, green fragrance reminiscent of freshly cut grass and acts as a defense signal to attract pollinators or predators of herbivores (Schwery et al., 2023). Heptanal, with its sweet, fruity, and slightly oily scent, appeals to nocturnal pollinators like moths and may repel herbivores (Unsicker et al., 2009). The genotype-dependent accumulation of *n*-octanal in ‘Carlos’ and ‘Late Fry’ cultivars, known for its citrusy and sweet fragrance, suggests a role in attracting pollinators such as bees and butterflies while also contributing to plant defense (Schwab et al., 2008).

### Ketones

Five ketones were identified in muscadine flowers; however, their accumulation seems to be genotype-dependent irrespective of flower type (**Supplementary Table S2**). Several muscadine flower genotypes exhibited undetectable ketones; however, the maximal total ketones production was identified in female flower genotype ‘O26-15-1’ with an average level of 1455 ± 247.4 µg/100g. The 6*Z*-pentadecen-2-one ketone was detected in perfect flower genotypes of ‘C11-2-2’. In the female flowers, the pentadecan-2-one was identified in ‘Onyx’, ‘O23-2-1’, and ‘Darlene’; however, ‘O23-2-1’ produces an extra ketone of 2-ethyl-5-methyl-1,3,2-dioxaborolan-4-one. The ketones of 2-pentadecanone and 2-nonadecanone were detected in both flower types (**Supplementary Table S2**). The ketones identified in muscadine flowers contribute in distinct ways to the complexity of floral scent and the ecological interactions that support pollination and defense. Their presence and concentration appear to be genotype-dependent, with certain flower types exhibiting unique ketone profiles.

The compounds of 6*Z*-pentadecen-2-one and pentadecan-2-one provide subtle, waxy, and fatty odors that add depth and persistence to floral scents to attract pollinators such as bees and nocturnal insects over long distances. These less volatile ketones linger, making the fragrance effective throughout the day and night (Schwab et al., 2008; Gibernau et al., 2021). Similarly, 2-pentadecanone and 2-nonadecanone contribute a persistent, waxy scent, enhancing the complexity and duration of the floral fragrance, which boosts pollinator attraction while potentially signaling defense to herbivores (Unsicker et al., 2009). The boron-containing compound 2-ethyl-5-methyl-1,3,2-dioxaborolan-4-one, though less characterized, likely contributes to ecological interactions and plant defense (Gibernau et al., 2021).

### Miscellaneous

Four miscellaneous volatiles, including givaudan, linoleoyl chloride, methyl (6*Z*,9*Z*,12*Z*,15*Z*)-octadeca-6,9,12,15-tetraenoate, and 6,7-dimethyl-5,6,7,8-tetrahydrotetrazolo[1,5-b][1,2,4]triazine were identified in muscadine flowers, with these compounds being more abundant in perfect flowers. Linoleoyl chloride, an acyl chloride derivative of linoleic acid, serves as a precursor in the biosynthesis of various volatile compounds, such as aldehydes, esters, and alcohols, which contribute significantly to floral fragrances. Though it does not directly produce scent, its derivatives play a crucial role in floral signals that mediate plant interactions with their environment, balancing attraction and defense against herbivores (Dudareva and Pichersky, 2006). Methyl (6*Z*,9*Z*,12*Z*,15*Z*)-octadeca-6,9,12,15-tetraenoate, a methyl ester of linolenic acid, adds sweet, fatty, and fruity undertones to the overall floral fragrance. These methyl esters are important for long-distance scent dispersion and contribute to the plant´s chemical signaling pathways, attracting pollinators like bees and butterflies while also aiding in defense responses (Pichersky and Gershenzon, 2002). The nitrogen-containing heterocyclic compound 6,7-dimethyl-5,6,7,8-tetrahydrotetrazolo[1,5-b][1,2,4]triazine adds earthy or spicy qualities to floral scents. These nitrogenous volatiles play a distinct role in plant-pollinator and plant-herbivore interactions, offering a unique scent signature that can influence ecological dynamics by attracting or repelling insects (David and Doro, 2023).

### Acids

Only two acids were detected in muscadine flowers, where the female flowers genotype, ‘O23-2-1’, produced the 2,5-diamino-2-methyl pentanoic acid, while 3-decenoic acid was only identified in perfect flower cultivars ‘Floriana’ and ‘Carlos’ (**Supplementary Table S2**). The 2,5-diamino-2-methylpentanoic acid is not directly involved in scent production, but it could influence floral fragrance through its role in amino acid and nitrogen metabolism, indirectly contributing to the synthesis of volatile compounds (Dudareva et al., 2006). The 3-decenoic acid, on the other hand, may serve as a precursor to floral volatiles that contributes to the fruity, green, or waxy characteristics of flower scents, and it could also play a role in plant defense mechanisms (Pichersky and Gershenzon, 2002; Dötterl and Jürgens, 2005; Knudsen et al., 2006). Both compounds exemplify how primary metabolites are linked to the production of secondary metabolites, which are essential for ecological interactions and pollinator attraction.

### Hydrocarbon dominates the volatile profiling in muscadine flowers

**Figure 2:**
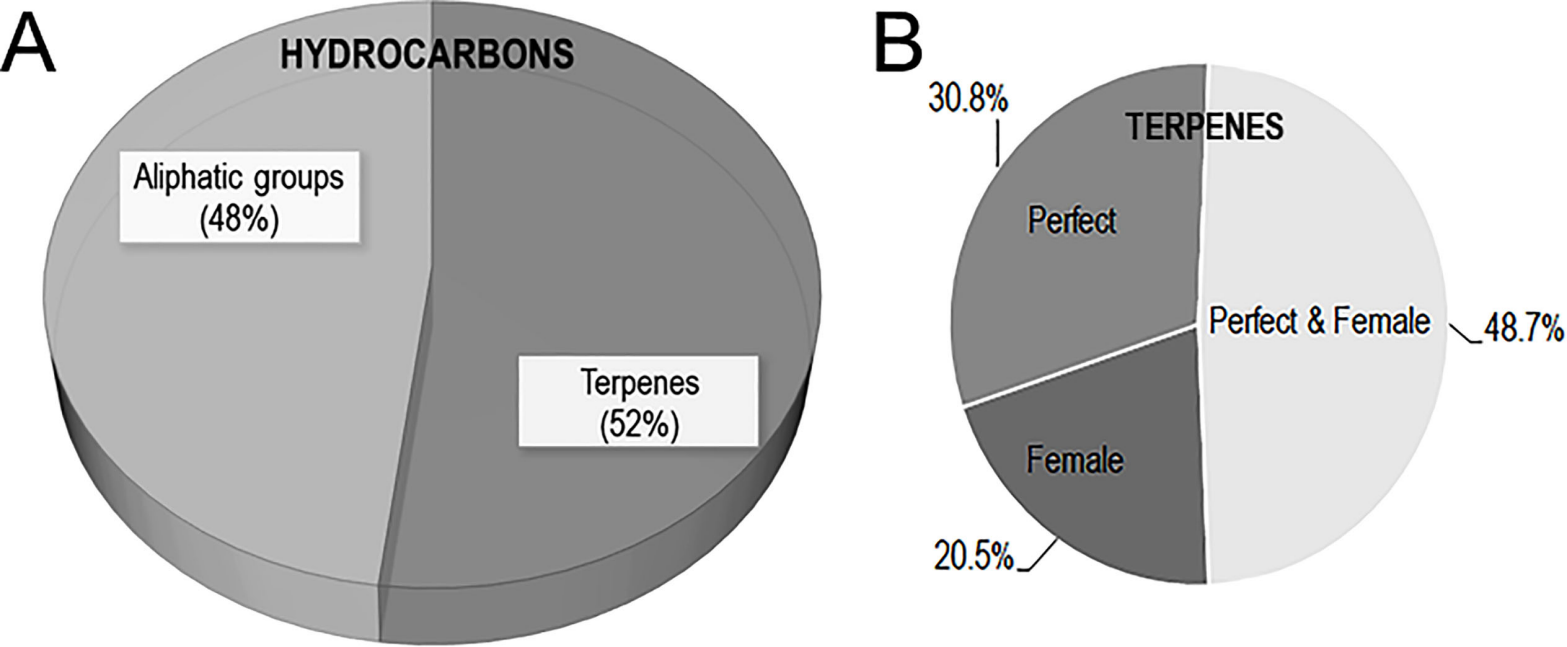
**(A)** Pie chart of hydrocarbon sub-class volatiles in muscadine flowers. **(B)** Pie chart of the distribution of terpenes volatiles in perfect and female muscadine flowers.

A total of seventy-seven (52.1%) hydrocarbons were detected in muscadine flowers (**Supplementary Table S2**). Among them, thirty-six (25%) volatiles were categorized under aliphatic compounds, and thirty-nine (27.1%) volatiles were classified under terpene volatiles based on their chemical properties (**Figure 2A**). Within the aliphatic groups, thirteen (36.1%) volatiles were exclusive to female flowers, seven (19.4%) volatiles were specific to perfect flowers, and sixteen (44.4%) were detected in both flower types. Overall, perfect flowers emitted a significantly higher number of aliphatic compounds (11.1 ± 1.1) compared to female flowers (7.8 ± 1.2). The female ‘Darlene’ flowers showed the lowest aliphatics number (3), while the perfect ‘Late Fry’ flowers emitted the highest (18), highlighting considerable variation in aliphatics production across different genotypes. The levels of total aliphatics produced by muscadine flowers considerably altered between genotypes and displayed a wide range with a minimum and maximum level of 122.5 ± 20.8 µg/100g (female flower ‘Darlene’) and 3110 ± 497.6 µg/100g (female flower ‘Majesty’), respectively. The *n*-tetradecane and (*E*)-9-octadecene were dominant in perfect flowers. In contrast, no unique aliphatic compound was identified for female flowers. On average, perfect flowers emitted ~1.4 times more aliphatic compounds than female flowers. The 5-methylundecane, 3-methylundecane, *n*-dodecane, *n*-nonadecane, *n*-heneicosane, *n*-docosane, and tricyclo[3.1.0.0(2,4)]hexane, 3,6-diethyl-3,6-dimethyl-, trans- were detected among different flower types (**Supplementary Table S2**). Aliphatic hydrocarbons are a diverse group of compounds that contribute to the overall floral scent profile, particularly in species like muscadine grapes. Although they tend to be less volatile than other VOCs, they play a critical role in the structure and persistence of floral aromas. These hydrocarbons provide subtle background notes, such as waxy, oily, or occasionally woody scents, which complement and enhance the more prominent floral fragrance compounds. The primary functional roles of these aliphatic hydrocarbons include contributing to the physical stability of the flower, helping to prevent water loss, and providing a barrier against herbivory and pathogens. By forming a protective layer, they help maintain the structural integrity of the flower, which is essential for its reproductive success (Kapoor et al., 2023). Beyond their protective functions, aliphatic hydrocarbons influence pollinator behavior by stabilizing the emission of more volatile, attractive compounds, thus extending the duration of floral scent in the air. This extended-release can increase the flower’s attractiveness to pollinators, particularly those that rely on long-range scent detection, such as bees, moths, and butterflies. These insects are drawn to flowers that maintain a consistent olfactory signal over time, helping them to navigate toward the floral source (Paré and Tumlinson, 1999; Jürgens, 2004; Junker and Blüthgen, 2010). Thus, while aliphatic hydrocarbons may be less prominent in odor, their ecological and functional roles are crucial for both the plant’s defense mechanisms and its interactions with pollinators.

Among 39 terpene volatiles identified in muscadine flowers, eight (20.5%) were found exclusively in female flowers, twelve (30.8%) were specific to perfect flowers, and nineteen (48.7%) were detected in both flower types (**Figure 2B**). Female flowers produced a significantly lower number of terpene volatiles (9.8 ± 1.2) than perfect flowers (13.5 ± 1.3). Among the evaluated flower types, the perfect ‘Late Fry’ flowers stood out with the highest terpene production, generating a total of 19 distinct terpenes. In contrast, the female ‘Darlene’ flowers exhibited the lowest terpene number, with only 4 terpenes identified. This variation in terpene production highlights the differences in volatile compound profiles between various flower types and genotypes, suggesting that the genetic background and flower structure may influence the biosynthesis of these important aromatic compounds. Understanding these differences can provide valuable insights into the factors that contribute to flower scent and, consequently, pollinator attraction. The total terpene levels in muscadine flowers varied widely, ranging from 51.4 ± 7.3 µg/100g in female ‘C8-6-1’ flowers to 4072.8 ± 576.6 µg/100g in perfect ‘B20-18-2’ flowers. Notably, the sesquiterpenes (*Z*,*E*)-α-farnesene and α-selinene, along with the diterpenoid alcohol (*E*,*E*)-geranyl linalool, were exclusively detected in female flowers (**Supplementary Table S2**). In contrast, four terpenes, including β-elemene, ɤ-elemene, α-muurolene, and β-bisabolene, were unique to perfect flowers. Several terpenes, though present in both flower types, exhibited significantly higher accumulation levels in perfect flowers. These included selina-4,11-diene (~19.8-fold), selina-4(15),7(11)-diene (~4.4-fold), valencene (~8.4-fold), (*E*)-β-farnesene (~2.6-fold), α-farnesene (~3.3-fold), germacrene D (~5.6-fold), junenol (~2.4-fold), and (–)-kaurene (~6.4-fold). Among these, valencene has been identified as an effective floral volatile in *Vitis* species, primarily localized in the anthers and pollen grains (Martin et al., 2009). This compound is also a key active terpene in orchid flowers, where it plays a crucial role in attracting Euglossine bees (Gerlach and Schill, 1991). Similarly, α-farnesene is a major terpene emitted by butterfly bush (*Buddleja davidii*) flowers, eliciting strong antennal responses in butterflies and moths (Andersson, 2003; Guédot et al., 2008). Likewise, (*Z*,*E*)-α-farnesene has been shown to stimulate antennal responses in honeybees (Dötterl and Vereecken, 2010).

Terpene volatiles are integral to floral scent profiles and serve vital ecological functions, including attracting pollinators and deterring herbivores. Muscadine floral terpenes, which include sesquiterpenes, diterpenoid alcohols, and monoterpenoid alcohols, contribute a range of aromas from fruity and floral to woody and earthy. These terpenes are particularly important for the short- and long-range attraction of pollinators. This diversity adds complexity to the floral bouquet, enabling individual flowers to stand out to pollinators. Most muscadine terpenes, significantly contribute to defining floral scent profiles and play dual roles in both attracting pollinators and defending against herbivores by making the plant less palatable, attracting predators of herbivores, or repelling herbivores or pathogens (Pichersky and Gershenzon, 2002; Dudareva et al., 2006; Knudsen et al., 2006; Junker and Blüthgen, 2010; Schiestl, 2010). The floral organs, petals, stamens, pistils, sepals, and nectaries, are the primary sources of floral volatiles in many plant species (Dobson et al., 1996). However, pollen plays a critical role in pollinator attraction, producing substantial quantities of volatiles distinct from those emitted by other floral organs (Dobson and Bergstroem, 2000). In *Vitis* species, sesquiterpene biosynthesis has been localized specifically within the anthers and developing pollen grains (Martin et al., 2009). This suggests that volatile emissions decrease after pollination, signaling the availability of pollen to pollinators. Since pollen is a major reward for pollinators, its quantity and viability significantly influence pollinator visits (Carr et al., 2015). Consequently, female flowers, which lack pollen, may suffer from reduced pollinator attraction due to lower terpene emissions and the absence of the pollen reward. Perfect muscadine flowers exhibited ~4.5-fold higher terpene production compared to female flowers. This enhanced production likely gives perfect flowers a competitive edge in attracting pollinators.

### Cross-correlation analysis among muscadine variables

The relationships between the accumulation of different VOCs and the type of muscadine flowers were examined using the Pearson correlation coefficient. Results revealed a strong positive correlation between the number of flowers per inflorescence and terpene level (r² = 0.75, *P* = 8.5 × 10^-4^), suggesting that the higher terpene output in perfect flowers may enhance pollinator attraction compared to female flowers. This higher terpene production in perfect flowers is likely due to the presence of pollen, which has been shown to influence VOC levels (Carr et al., 2015).

Moreover, the accumulation of the aldehydes was positively correlated with esters (r² = 0.84, *P* = 5.5 × 10^-5^), terpenes (r² = 0.73, P = 1.4 × 10^-3^), aliphatic (r² = 0.77, *P* = 5.1 × 10^-4^), and miscellaneous levels (r² = 0.87, *P* = 1.4 × 10^-5^). Esters accumulation was also positively correlated with terpenes (r² = 0.71, *P* = 1.9 × 10^-3^), aliphatic (r² = 0.76, *P* = 7.3 × 10^-4^), and miscellaneous compounds (r² = 0.75, *P* = 8.6 × 10^-4^). These correlations can be due to the overlapping biosynthetic pathways, co-regulation by similar environmental factors, synergism effects due to the combination of VOCs, and/or their complementary contributions to the floral attractive and complex scents (Pichersky and Gershenzon, 2002; Dudareva et al., 2006; Knudsen et al., 2006; Pichersky et al., 2006; Tholl et al., 2006). Such coordinated production allows the maximum effect of the flower on the pollinators and plant protection from herbivores. Besides, a significant correlation was observed for terpenes and aliphatic hydrocarbons (r² = 0.52, *P* = 3.8 × 10^-2^). Both terpenes and aliphatic hydrocarbons originate from the common pool of primary metabolites. Aliphatic hydrocarbons are derived from the fatty acid metabolite while terpenes are produced via the mevalonate or methylerythritol phosphate pathways, starting from acetyl-CoA. The production of terpenes and aliphatic hydrocarbons can be associated with the plant’s metabolic state. Increased flux of primary metabolites as acetyl-CoA can be beneficial for enhancing the production of both classes of compounds (Dudareva et al., 2013).

### Multivariate statistical analysis of terpene volatiles in muscadine flowers

**Figure 3:**
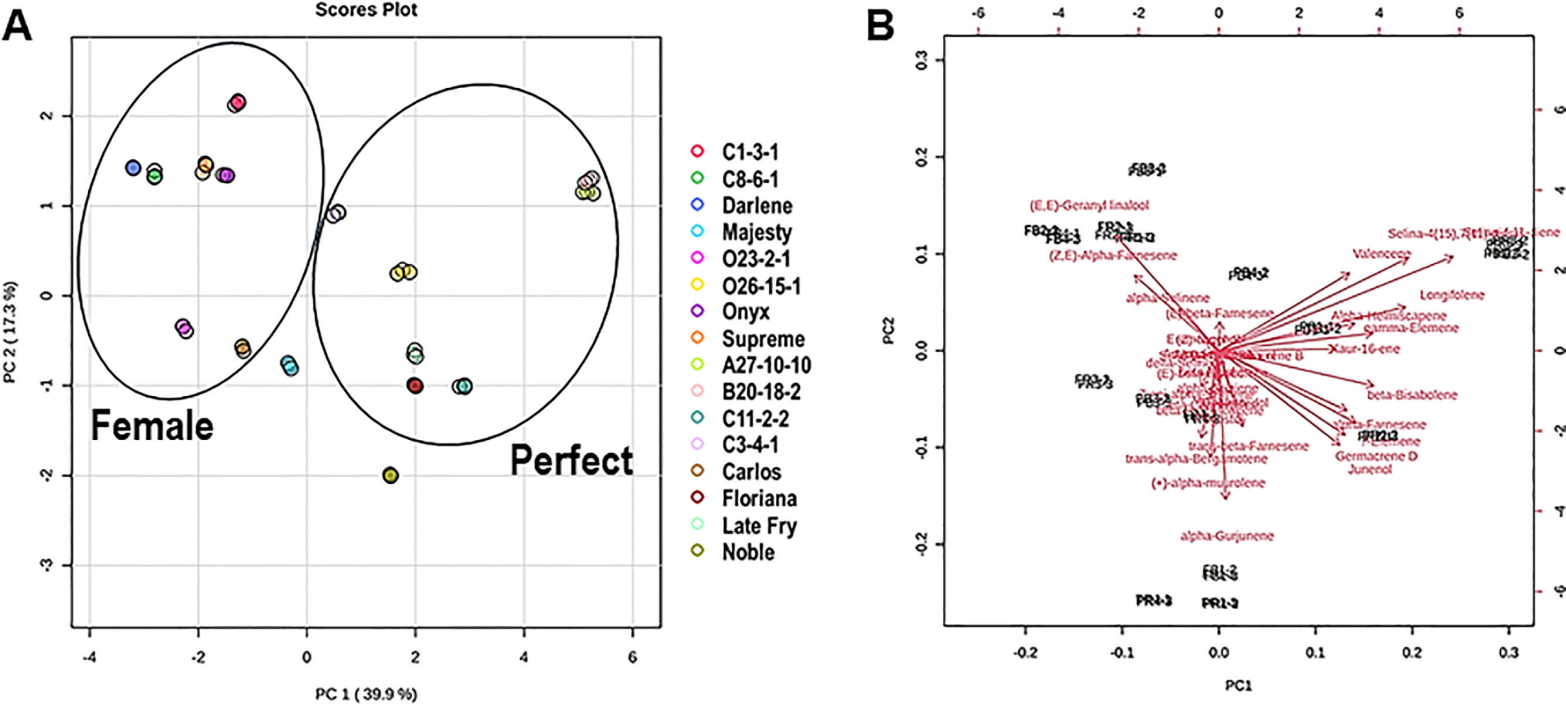
**(A)** Principal component analysis (PCA) 2D score plot and **(B)** Biplot of terpenes in different muscadine flowers. In the 2D score plot, the several colors and shapes represent muscadine flower genotypes. The scores of the observations (i.e., muscadine flower genotypes) are indicated. The vectors that point toward the same direction correspond to the variables (i.e., volatile metabolites) with similar response profiles.

Multivariate statistical analysis was performed using the terpene volatiles detected in muscadine flowers to determine the potential differences between female and perfect flowers. Terpenes were chosen due to their key roles in pollinator attraction, floral scents, and biological relevance (Crowell et al., 2002; Farré-Armengol et al., 2017; Qiao et al., 2021). Principal component analysis (PCA) revealed that the first two components accounted for 39.9% and 17.3% of the variance, respectively (**Figure 3A**). Perfect and female flowers deviated from each other and clustered depending on their similarities in the terpene volatile context. However, certain genotypes, including the perfect ‘Noble’ and female ‘Majesty’ flowers, were located out of the two groups, suggesting that their terpene accumulation is more genotype-dependent than flower-type-dependent. PCA biplot analysis also displayed the separation of flowers’ type and their close aggregation based on their similarities in composition (**Figure 3B**). The (*Z*,*E*)-α-farnesene and (*E*,*E*)-geranyl linalool were computed as dominant contributors to the cluster of female flowers. At the same time, the selina-4,11-diene, selina-4(15),7(11)-diene, and valencene were the primary volatile terpenes contributing to the perfect flower group.

**Figure 4:**
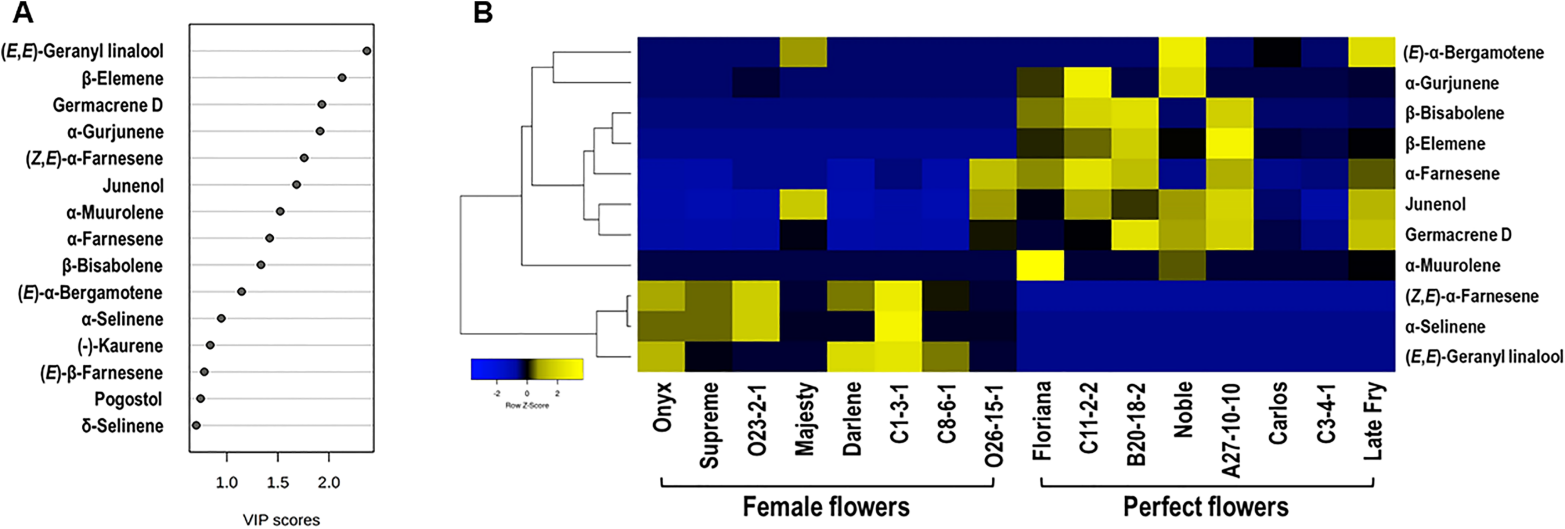
**(A)** Variable importance in projection (VIP ≥ 1.0) measure in PLS-DA analysis. **(B)** Hierarchical Clustering Heatmap of VIP (score ≥ 1.0) terpenes in muscadine flower genotypes. In Heatmap analysis, each column refers to the muscadine flower genotype, and each row indicates the marker volatile terpenes. The blue and yellow colors with values (ranging from –2 to +2) describe the lower and higher terpene intensities; the higher the yellow color intensity (> 0 to +2 values), the higher terpene levels. In contrast, the higher blue color intensity (< 0 to –2 values) represents the lower terpene levels.

To further assess the diversity in terpenes among muscadine flowers, a supervised partial least squares-discriminant analysis (PLS-DA) was performed. The analysis identified key terpenes based on variable importance in projection (VIP) scores. Eleven volatile terpenes with VIP scores above 1.0 were identified, including (*E*,*E*)-geranyl linalool, β-elemene, germacrene D, α-gurjunene, (*Z*,*E*)-α-farnesene, junenol, α-muurolene, α-farnesene, β-bisabolene, (*E*)-α-bergamotene, and α-selinene (**Figure 4A**).

Floral terpenes are volatile compounds that play pivotal roles in plant development, defense, and pollinator attraction (Degenhardt et al., 2009). These molecules serve a variety of ecological functions, from attracting pollinators to providing direct and indirect protection against insects, bacteria, and fungi (Gershenzon and Dudareva, 2007; Boncan et al., 2020). To examine the differences in terpene profiles between muscadine flowers, a heatmap was generated using Pearson correlation analysis, illustrating the relationships between the relative levels of key volatile terpenes and each flower genotype (**Figure 4B**). The analysis identified both positive and negative correlations, with the eleven volatile metabolites emerging as key marker terpenes. These markers effectively differentiate female and perfect flowers. Among them, five shared terpene markers were found to be significantly higher in perfect flowers (~4.7-fold), highlighting the distinct variation in terpene context between the two flower types. Of these, only two terpenes, the sesquiterpenes α-farnesene (r² = 0.71, *P* = 1.9 × 10^-3^) and germacrene D (r² = 0.73, *P* = 1.4 × 10^-3^), showed a significant positive correlation with perfect flowers, suggesting their critical role in defining floral scent and ecological interactions. Both α-farnesene and germacrene D are integral components of floral scents, contributing to their complexity and appeal (Mostafa et al., 2022). Their unique aromatic profiles, along with their interactions with other volatile compounds, enhance the overall fragrance of flowers, making them attractive to pollinators and vital for plant reproduction. Understanding their roles in flower odor provides valuable insights into the ecological dynamics between plants and their pollinators.

The different flower organs generally contribute to the overall floral volatiles (Dobson et al., 1996). Female and perfect flowers display similar structures, excluding stamens and pollen. Pollens produce considerable amounts of floral volatiles that serve as master attractants for pollinators, and they can be easily distinguished from the scents of other floral organs due to their remarkable effectiveness (Dobson and Bergstroem, 2000; Martin et al., 2009). It is tempting to suggest that the difference in terpene quality between female and perfect flowers (i.e., type, number, and quantity) may be due to pollen presence. Three terpene markers, β-elemene, α-muurolene, and β-bisabolene, were exclusively accumulated in perfect flowers. The β-elemene sesquiterpene has been previously identified as a marker volatile for pollen odor in bay laurel (*Laurus nobilis*) flowers (Flamini et al., 2002). Known for its unique odor profile, β-elemene contributes spicy, woody, and citrusy notes to floral fragrances, enhancing the complexity and distinctiveness of flower aromas (Knudsen et al., 2006; Raguso, 2008). Variations in β-elemene content across species and within genotypes create distinct olfactory profiles that attract specific pollinators. Its presence in flowers like *Cymbidium* orchids and *Rosa* species further underscores its significance in shaping fragrance landscapes (Dobson, 2006; Farré-Armengol et al., 2017). Additionally, β-elemene facilitates ecological interactions by attracting pollinators and providing indirect defenses against herbivores (Dudareva et al., 2006; Zhang, 2018). Similarly, α-muurolene serves as a distinctive marker for perfect flower genotypes. This sesquiterpene, known for its woody, earthy, and slightly spicy scent, enhances the olfactory signatures of flowers. Identified in the volatile emissions of several flowers, including *Rosa* and *Lilium* species, α-muurolene adds depth to floral aromas and aids in differentiating flowers based on their scent profiles (Knudsen et al., 2006). It plays a crucial ecological role, acting as an attractant for pollinators while deterring herbivores and pathogens. By adding earthy undertones, α-muurolene can enhance the appeal of flowers to specific insect visitors (Dudareva et al., 2013). Its presence often complements other terpenes, contributing to the overall complexity of the floral bouquet and distinguishing species or varieties with pronounced earthy or resinous scents (Jürgens et al., 2003; Raguso, 2008). Finally, β-bisabolene is pivotal in distinguishing floral fragrances due to its characteristic woody, spicy, and mildly sweet scent. This compound enriches the olfactory profile of certain plant species and plays a dual role in attracting pollinators while providing defensive properties against herbivores and pathogens through its antimicrobial and insect-repellent activities (Gershenzon and Dudareva, 2007; Maffei et al., 2011). Its exclusive presence in perfect flowers emphasizes its role in differentiating floral types within the species, contributing to the richness of the terpene blend (Degenhardt et al., 2009).

Another set of three terpenes, (*Z*,*E*)-α-farnesene, (*E*,*E*)-geranyl linalool, and α-selinene, was found exclusively in female muscadine flowers. The (*Z*,*E*)-α-farnesene is particularly significant due to its contribution to the scent profiles of various flowers, including jasmine, where it imparts sweet, floral, and fruity characteristics (Zhang et al., 2021). This sesquiterpene interacts synergistically with other volatile compounds, such as linalool and geraniol, enhancing the overall fragrance profile (Murray et al., 2024). Additionally, its appealing fruity aroma attracts pollinators, thus playing a crucial role in plant reproduction (Wang and Erb, 2022). The (*E*,*E*)-geranyl linalool is a floral compound that possesses a unique structure that includes both geranyl and linalool moieties, allowing it to emit a complex sweet and slightly citrusy scent. It works synergistically with other volatile compounds to enhance the overall fragrance, resulting in a more rounded and complex floral scent (Xu et al., 2021). Its presence in floral scents can signal the availability of nectar, thus attracting essential pollinators and promoting plant reproduction (Raguso, 2008). Studies have shown that specific floral fragrances, including those containing (*E*,*E*)-geranyl linalool, can influence pollinator behavior, leading to increased visitation rates and enhancing the success of fertilization (Knudsen et al., 2006). On the other side, the female flower-exclusive α-selinene, while enhancing floral aromas, has been reported as a repellent sesquiterpene that negatively affects bee attraction in other plant species (Quarrell et al., 2023). In muscadine, such emissions may compromise female flowers’ ability to attract pollinators.

VOCs facilitate the interaction between plants and mutualists, pests, and pathogenic antagonists. Semiochemicals that function in these interactions can be produced constitutively or in response to outside interactions and stimuli that occur above and below ground (Massalha et al., 2017). Although semiochemicals can travel long distances, plant/plant and plant/microbe communication usually occurs at relatively short distances, while plant volatiles with a role in plant/insect interaction are perceived at long distances. The resulting extreme dilution and the significant variation in chemical structures and properties of the VOCs pose a challenge to the analysis of the volatiles and their precursors (Fu et al., 2017). Floral terpenes are widely classified based on the disparity in composition, amount, and emission (Muhlemann et al., 2014). They are further categorized based on their scent spread efficiency to long- and short-distance terpenes, targeting specific or broad pollinators (Guterman et al., 2002; Schiestl et al., 2003). These terpene volatiles can effectively attract different pollinators such as bees, lovebugs, ants, and wasps (Bouwmeester et al., 2019). The composition of muscadine floral volatiles is complex and diverse. It may be challenging for the pollination vectors to identify their flower hosts quickly and accurately in a complex environment if relying solely on the attractiveness of a specific floral volatile compound. Accordingly, it is tempting to speculate that the identified floral terpene volatile markers are potentially coordinated to attract long-range and short-range localized pollinators, ensuring appropriate fruit set efficiency.

### Determination of fruit set efficiency in female and perfect flowers

**Figure 5:**
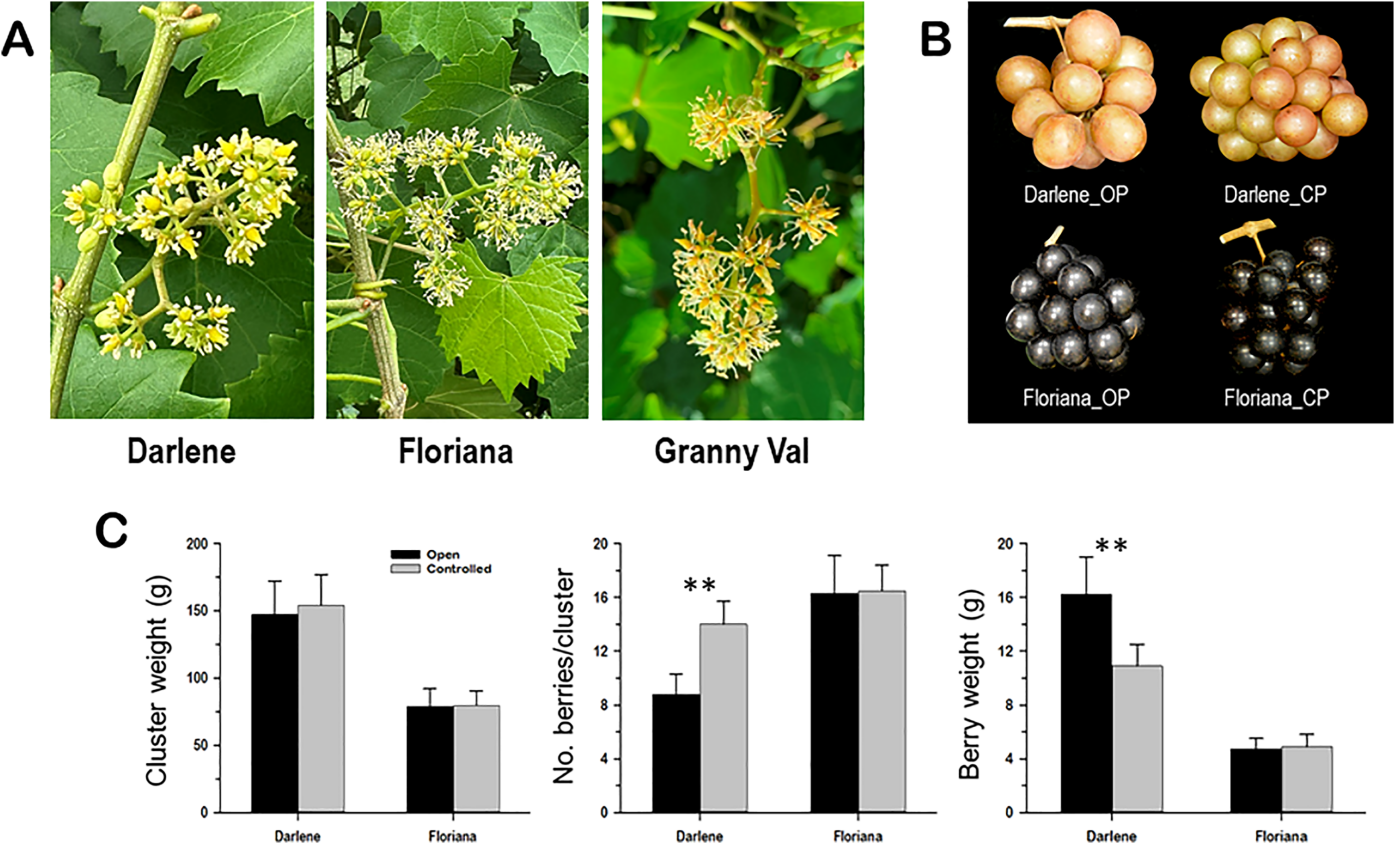
**(A)** Close-up views of the female 'Darlene' and perfect 'Floriana' muscadine flowers used for the fruit set efficiency assay; however, the perfect ´Granny Val´ flowers were used as a source of pollens. **(B)** Close-up views of 'Darlene' and 'Floriana' ripe clusters resulted from open pollination (OP) and controlled pollination (CP). **(C)** Determination of the capacity of perfect and female flowers to set berries in response to open and controlled pollination. The characters of cluster weight (g), number of berries/cluster, and berry weight (g) were used for the assessment. Data represent the mean values ± SD (*n* = 5). ** Significant at p ≤ 0.01.

Our results suggested that perfect flower muscadine genotypes potentially exhibit a greater pollinator attraction capacity than female flowers, likely due to the superior quality of terpenes produced. The interaction between flowers and insect pollinators can be assessed by evaluating the efficiency of flower-to-fruit set progression within the inflorescence. To explore this further, we assessed the fruit set capacity of both female and perfect flowers under two pollination conditions: open pollination (insect-dependent) and controlled pollination. The female cultivar ‘Darlene’ and the perfect flower cultivar ‘Floriana’ were selected for the study, with pollen from the perfect flower cultivar ‘Granny Val’ used in the controlled pollination experiments (**Figure 5A**).

Controlled pollination significantly altered fruit set efficiency and cluster characteristics in ‘Darlene’ but not in ‘Floriana’ (**Figure 5B**). In ‘Darlene’ controlled pollination resulted in very compact clusters, driven by a ~60% increase in the number of berries per cluster, coupled with a ~33% reduction in individual berry weight, leading to only minor changes in total cluster weight (**Figure 5C**). Interestingly, no significant changes in fruit set efficiency or cluster characteristics were observed in ‘Floriana’ under controlled pollination.

These results suggested that female muscadine flowers may not reach their full fruit set potential due to lower pollinator attraction efficiency, leading to sporadic pollinator visits. When this limitation was eliminated through hand pollination, fruit set accuracy improved significantly. In contrast, perfect flower muscadines do not perceive this challenge, as they are self-pollinated, and supported by a well-developed floral volatilome machinery that attracts pollinators.

## Conclusions

**Figure 6:**
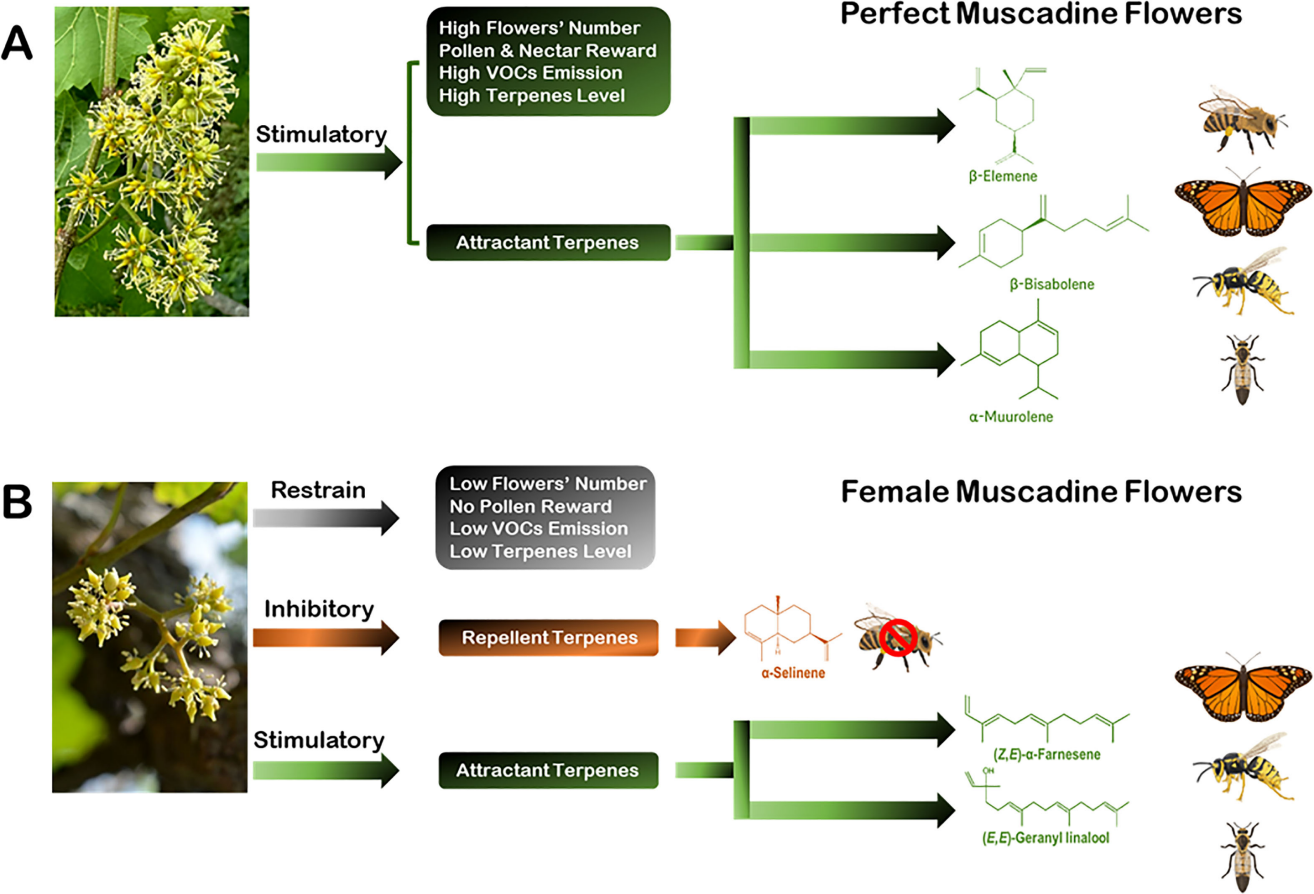
Descriptions of the characteristics of perfect and female muscadine flowers highlight several parameters influencing their ability to attract pollinators. These parameters encompass floral morphology, rewards, the emission of VOCs, and specific terpenoid profiles. Such traits play a vital role in drawing diverse pollinators, including butterflies, lovebugs, wasps, and bees. **(A)** Perfect muscadine flowers exhibit notable advantages for pollinator attraction through a combination of favorable attributes. They are characterized by a high number of flowers, abundant pollen, nectar availability, and elevated levels of effective VOCs and terpenes. These features work synergistically to support efficient and reliable pollination processes. **(B)** By contrast, female muscadine flowers show a reduced prevalence of these floral traits. They are defined by a lower flower count, the absence of pollen, and diminished levels of attractive VOCs and terpenes. Furthermore, female flowers emit volatile terpenes, such as α-selinene (indicated in orange), which may deter bees instead of attracting them. However, certain volatile compounds associated with pollinator stimulation, marked in green, still showcase their capacity to draw in other visitors, albeit to a lesser extent.

The dynamic of plant/insect pollinator interaction is a bi-directional communication procedure. Consequently, many mechanisms have evolved to engage organisms in different types of interactions. Flower factors, including morphological parameters, reward potentials, and allelochemicals context coordinate the dialogue between flowers and pollinators. Allelochemicals, mainly volatile terpenes, can mediate these critical interactions. However, the interaction mechanism and the kind of vector pollinator depend on the context of terpenes and VOCs emitted in terms of style, number, intensity, function, and environmental circumstances. The self-pollinated perfect muscadine flowers are distinguished from the obligatory cross-pollinated female flowers by possessing several floral attributes, including visual (i.e., flower number), reward (i.e., pollen and nectar), and biochemical (i.e., VOCs and terpenes) parameters to ensure an accurate pollination procedure (**Figure 6A**). The prevalence of these floral parameters was, to some extent, compromised in female flowers, but even more, female flowers emit volatile terpenes that contradict pollinators rather than attract them (**Figure 6B**). The development of the knowledge on species-dependent floral parameters, particularly VOCs emitted by flowers is of great importance for plant ecology in the context of environmental and climate changes. The findings in this study show that floral scent plays an important role in structuring flower–insect relations in complex and challenging environmental circumstances. Understanding the effect of flower sex patterns in floral VOC profiles may have important implications for plant-pollinator interactions among communities differing in species composition.

## Data Availability

The original contributions presented in the study are included in the article/**Supplementary Material**. Further inquiries can be directed to the corresponding author.
