# Supplementary material for: Untargeted flower volatilome profiling highlights differential pollinator attraction strategies in muscadine
Source: Front Plant Sci. 2025 Feb 28;16:1548564. doi: 10.3389/fpls.2025.1548564 (PMC11906380; doi:10.3389/fpls.2025.1548564)
Supplement: Supplementary file 1 [file DataSheet1.pdf]

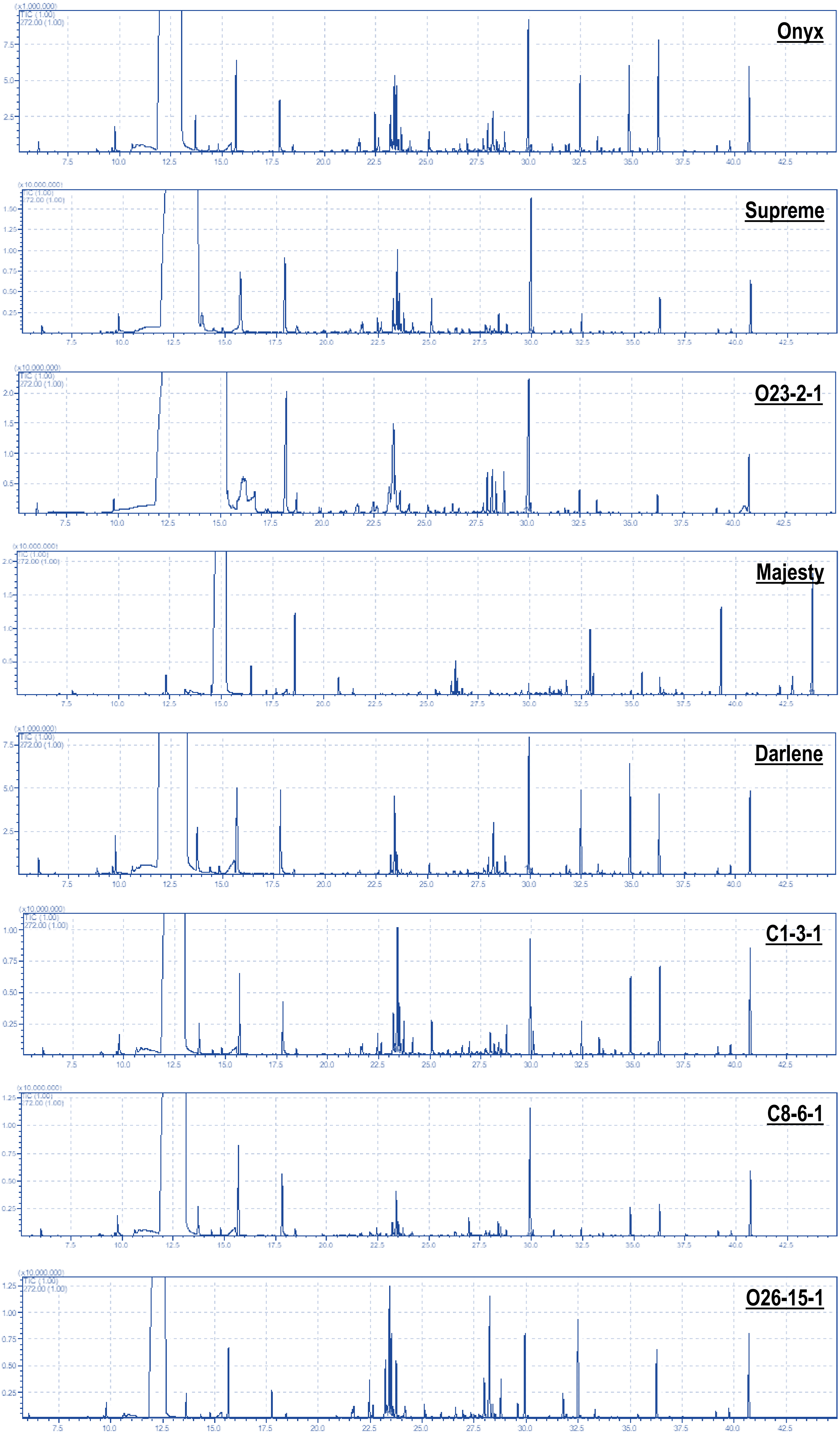
Onyx
Supreme
O23-2-1
Majesty
Darlene
C1-3-1
C8-6-1
O26-15-1

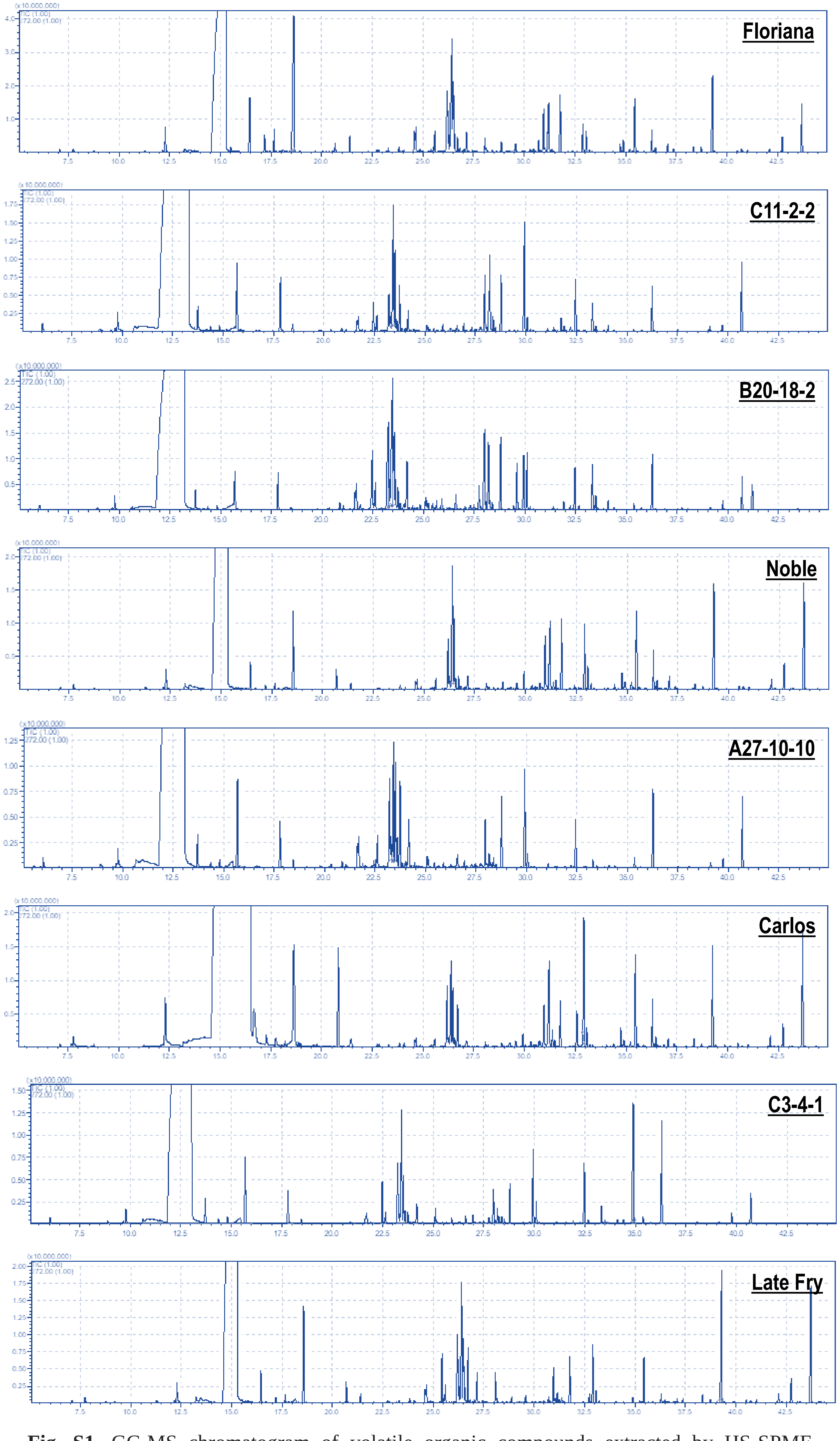

Fig. S1. GC-MS chromatogram of volatile organic compounds extracted by HS-SPME from different muscadine flower genotypes. The name of muscadine cultivar or breeding line is indicated in each figure.
