# Supplementary material for: Untargeted flower volatilome profiling highlights differential pollinator attraction strategies in muscadine
Source: Front Plant Sci. 2025 Feb 28;16:1548564. doi: 10.3389/fpls.2025.1548564 (PMC11906380; doi:10.3389/fpls.2025.1548564)
Supplement: Supplementary file 2 [file Table1.docx]

| **Table S1:** List of muscadine genotypes used in this study along with parental pedigree | | | | | |
| --- | --- | --- | --- | --- | --- |
| **Genotype** | **Herbarium No.** | **Female Parent** | **Male Parent** | **Type of Flower** | **Berry Color** |
| Onyx | CVSFR A411 | Supreme | Ison | Female | Purple |
| Supreme | CVSFR O421 | Black Fry | Dixieland | Female | Purple |
| O23-2-1 | CVSFR O2321 | Majesty | Tara | Female | Purple |
| Majesty | CVSFR B411 | Black Fry | Dixieland | Female | Purple |
| Darlene | CVSFR O181 | 5-11-3 | Carlos | Female | Bronze |
| C1-3-1 | CVSFR C131 | Pam | Carlos | Female | Bronze |
| C8-6-1 | CVSFR C861 | Supreme | Tara | Female | Bronze |
| O26-15-1 | CVSFR O26151 | Fry | Granny Val | Female | Bronze |
| Floriana | CVSFR C1611 | Supreme | Pineapple | Perfect | Purple |
| C11-2-2 | CVSFR C1122 | Fry | Tarheel | Perfect | Purple |
| B20-18-2 | CVSFR B20182 | Fry | Tarheel | Perfect | Purple |
| Noble | CVSFR O321 | Thomas | Tarheel | Perfect | Purple |
| A27-10-10 | CVSFR C13152 | Pam | Granny Val | Perfect | Bronze |
| Carlos | CVSFR O161 | Topsail | Tarheel | Perfect | Bronze |
| C3-4-1 | CVSFR C341 | Darlene | Triumph | Perfect | Bronze |
| Late Fry | CVSFR O2131 | Fry | Granny Val | Perfect | Bronze |
